# An Atypical Case of Microscopic Polyangiitis Associated With Myelodysplastic Syndrome and Complete Atrioventricular Block Mistaken for Infectious Pneumonia

**DOI:** 10.7759/cureus.48566

**Published:** 2023-11-09

**Authors:** Axel Sudria, Hervé Hyvernat, Paul Hannetel, Lucas Morand, Jean Dellamonica

**Affiliations:** 1 Service d'Hématologie, Hôpital Archet 1, Université Nice Sophia-Antipolis, Nice, FRA; 2 Service de Médecine Intensive Réanimation, Hôpital Archet 1, Université Nice Sophia-Antipolis, Nice, FRA; 3 Équipe 2 CARRES, UR2CA - Unité de Recherche Clinique Côte d'Azur, Université Côte d'Azur, Nice, FRA; 4 Laboratoire Central d'Anatomie et Cytologie Pathologiques (LCAP), Hôpital Pasteur 2, Université Nice Sophia-Antipolis, Nice, FRA

**Keywords:** anca-associated vasculitis (aav), microscopic polyangeitis, paroxysmal complete heart block, alveolar hemorrhage, myelodysplasic syndrome

## Abstract

The association between antineutrophil cytoplasmic antibody (ANCA)-associated vasculitis and hematologic malignancy has been previously described and remains a rare phenomenon (although potentially underdiagnosed). We report the case of an 81-year-old patient with myelodysplastic syndrome who was managed for an infectious-appearing pneumonia, which subsequently complicated into complete heart block and severe acute respiratory distress syndrome with a fatal outcome. The final diagnosis is severe hemorrhagic alveolitis due to ANCA-associated vasculitis meeting the criteria for microscopic polyangiitis. This article provides an opportunity to discuss the association between ANCA-associated vasculitis and hematologic malignancies and the adverse prognosis associated with it.

## Introduction

The antineutrophil cytoplasmic antibody (ANCA)-associated vasculitides (AAV) are characterized by inflammation of small-caliber blood vessels [1]. This entity comprises three different diseases: granulomatosis with polyangiitis (GPA), microscopic polyangiitis (MPA), and eosinophilic GPA (EGPA), with an estimated incidence in Europe of 8.5 per million, 4.7 per million, and 1.7 per million individuals per year, respectively [2]. Two specific types of ANCA antibodies have been described: anti-myeloperoxidase (MPO) antibodies, more often associated with MPA and EGPA, and anti-proteinase 3 (PR-3) antibodies, more specific to GPA. We describe a case of MPA that stands out due to its association with myelodysplastic syndrome (MDS) and the development of complete atrioventricular block (AVB).

This article was previously posted on the ResearchGate preprint server on October 2, 2023.

## Case presentation

An 81-year-old woman with a history of hypothyroidism, osteoporosis, and lower extremity deep vein thrombosis treated with rivaroxaban for the past four months was admitted to our intensive care unit (ICU) for acute respiratory distress syndrome. She had no history of arthralgia, rash, or sinusitis. One year ago, she had a baseline creatinine of 0.69 mg/dL (eGFR of 84 mL/min). Four months earlier, she had consulted a hematologist for pancytopenia, presenting with a hemoglobin level of 11 g/dL, a platelet count of 54 × 109/L, and a total leukocyte count of 2.3 × 109/L (1.14 × 109/L neutrophils and 0.84 × 109/L lymphocytes). Bone marrow aspiration revealed normal overall cellularity with discrete qualitative anomalies and a 5% blast count. The karyotype was normal. She progressively experienced general fatigue without fever.

Ten days before her admission to ICU, she presented to the emergency department complaining of nausea and vomiting. Due to significant asthenia, she had been treated with levofloxacin for five days for right laterobasal pneumonia confirmed by a chest X-ray. A physical examination revealed a temperature of 37.2°C, blood pressure of 125/108 mmHg, a pulse rate of 63 beats/min, peripheral blood oxygen saturation (SpO2) of 95% on room air, and a respiratory rate of 28 breaths/min. Wet crackles could be heard on auscultation of the right pulmonary base. The laboratory tests showed an inflammatory syndrome and renal insufficiency. There was no eosinophilia.

**Figure 1:**
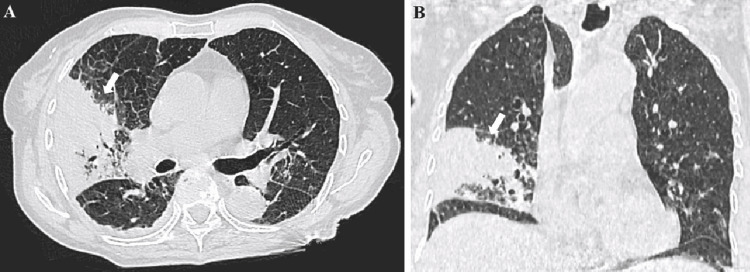
Unenhanced chest CT axial section (A) and sagittal section (B): consolidation of the middle lower lobe with air bronchogram and presence of several pseudonodular condensation areas

A thoraco-abdominal-pelvic scan revealed a right middle lobe consolidation and several pseudo-nodular consolidations, along with moderate hepatomegaly (arrow of 14 cm) (Figure 1).

**Figure 2:**
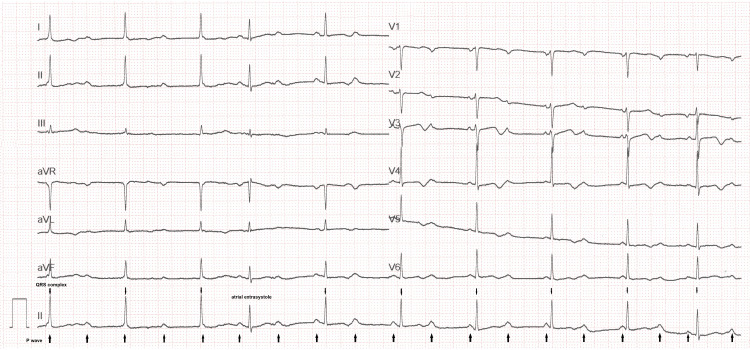
Complete atrioventricular block with sinus rhythm at 108 beats/min and junctional escape rhythm with narrow QRS complexes at 59 beats/min, suggestive of intranodal block

Legionella and pneumococcal antigenuria, as well as polymerase chain reaction testing for influenza A, influenza B, and COVID-19, all returned negative. The urine culture on the second stream was sterile, but cytology revealed leukocyturia (71 white blood cells/mL) and hematuria (65 red blood cells/mL). The troponin level was normal. A combination therapy with ceftriaxone and spiramycin was initiated, and she was admitted to the hospital. On the following day, she developed a well-tolerated complete AVB, which required the implantation of a pacemaker (Figure 2).

**Figure 3:**
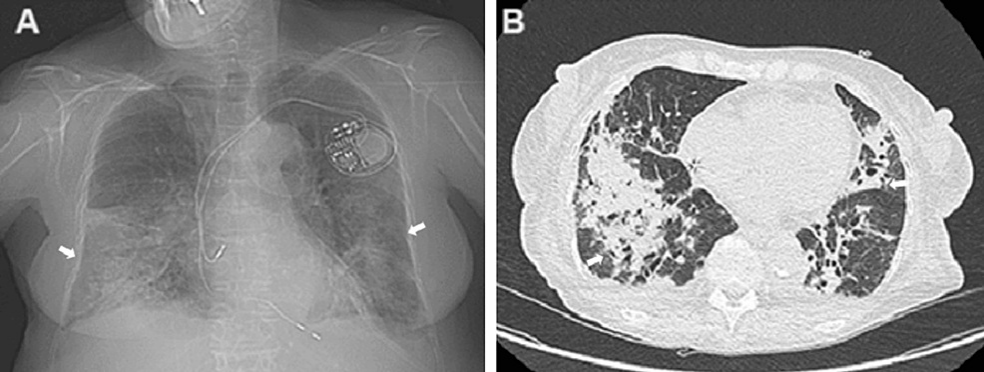
Chest X-ray: bilateral alveolar opacities predominantly at the right base (A); unenhanced chest CT scan, axial section: bilateral consolidations with air bronchogram and indistinct borders (B)

By the fourth day, she became reliant on oxygen at a rate of 4 L/min, without improvement in biological inflammation markers. She started treatment with piperacillin/tazobactam. By the seventh day, she experienced subconjunctival and oral bleeding without coughing up blood. Her hemoglobin levels dropped significantly to 7 g/dL, her white blood cell count increased substantially to 65 × 10^9^/L, and her platelet count decreased to 28 × 10^9^/L. She received transfusions of platelets and red blood cells. A repeat CT scan showed that the lung lesions were worsening (Figure 3).

On the ninth day, her respiratory condition deteriorated suddenly, and she required high-flow oxygen and transfer to the ICU. Upon admission, acute pulmonary edema was ruled out with a transthoracic ultrasound, and she was intubated quickly. The biological tests revealed severe ARDS associated with profound anemia and a significant increase in leukocytosis (Table 1).

**Table 1:** Laboratory values at admission to the emergency department and in the intensive care unit (ICU) (baseline creatinine = 0.69 mg/dL a year ago)

	Emergency	ICU	Reference range
Platelet count	110	28	150-400 × 10^9^/L
Hemoglobin	10.6	5.5	11.9-14.5 g/dL
White blood cells	14.7	78	4-10 × 10^9^/L
Neutrophils	12.8	60.8	1.8-7.2 × 10^9^/L
Lymphocytes	0.5	0.8	1.4-4.0 × 10^9^/L
Monocytes	1.4	7.0	0.2-1.0 × 10^9^/L
Metamyelocytes	0	6	0%
Myelocytes	0	5	0%
Promyelocytes	0	1	0%
Blasts	0	0	0%
C-reactive protein	227	114.7	<10 mg/L
Procalcitonin	1.59	2.37	<0.6 ng/mL
Creatinine	1.84	2.4	0.51-0.95 mg/dL
Urea	11.7	18	2.9-8.2 mmol/L
Troponin	9.7		<47.0 ng/L
Oxygen	Room air	100%	
pH	7.48	7.16	7.31-7.42
pO2	66.1	78.2	>60 mmHg
pCO2	27.2	81.2	32.0-45.0 mmHg
Lactate	0.9	7.4	0.0-1.3 mmol/L

**Figure 4:**
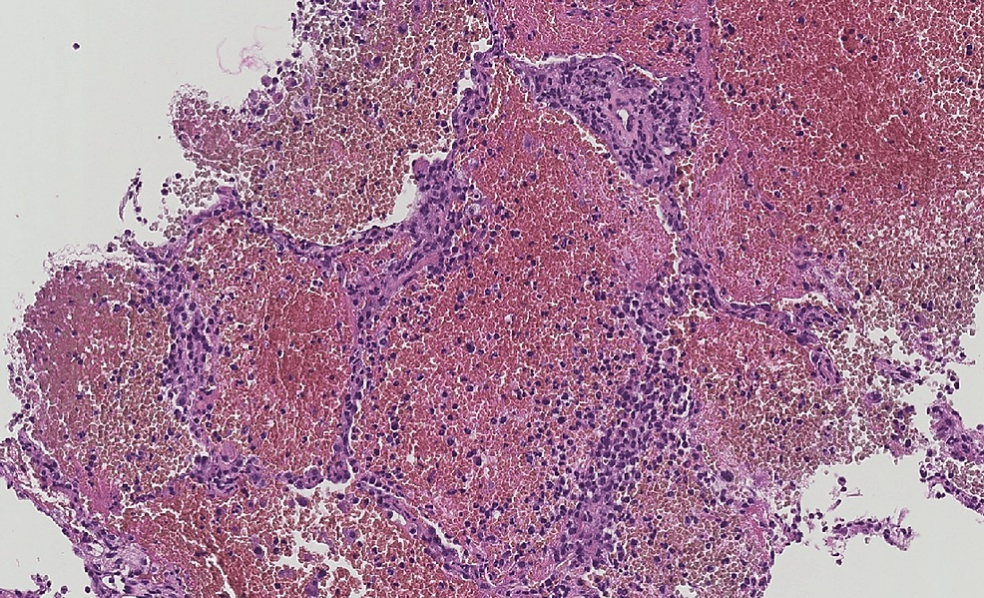
Lung biopsy (hematein-eosin stained) showing alveolar spaces filled by red blood cells, associated with mild alveolar wall thickening; magnification 17×

Protected distal aspiration revealed only a few red blood cells. Hemodynamic instability developed, accompanied by lactic acidosis, which required increasing doses of norepinephrine. The situation was further complicated by KDIGO stage 3 acute kidney injury and shock liver, ultimately resulting in her death in less than 24 hours. Retrospectively, respiratory and blood cultures all remained sterile. Blood tests revealed strongly positive anti-MPO antibodies (titer > 134 IU/ml), whereas anti-PR3, anti-glomerular basement membrane, and antinuclear antibodies were negative. A repeat bone marrow examination on admission found supporting evidence for an MDS: dyserythropoiesis (megaloblastic appearance and occasional basophilic punctuations in erythroblasts) and dysgranulopoiesis (granulated myeloid clone with chromatin condensation abnormalities) along with toxic granulations. The medullary karyotype was normal. Testing for BCR-ABL rearrangement by FISH and PCR was negative. Molecular analysis of the bone marrow (90-gene panel) contributed to the findings of three mutated genes: CBL, TET2, and PHF6. A post-mortem lung biopsy revealed severe lesions of hemorrhagic alveolitis with minimal leukocyte infiltration, confirming a diagnosis of intra-alveolar hemorrhage (IAH) (Figure 4).

## Discussion

AAV typically presents with renal involvement, ranging from urinary abnormalities to rapid glomerulonephritis, often accompanied by respiratory symptoms. Our case was diagnosed as MPA based on the ACR/EULAR 2022 criteria, which include high levels of perinuclear ANCA of the MPO type, the presence of IAH, and the absence of facial sinus abnormalities [3]. While anti-MPO antibodies are found in around 90% of MPA cases, they are also present in about 15% of granulomatous polyangiitis (GPA) cases [4]. Regrettably, our patient’s severe renal involvement, indicated by hematuria and renal insufficiency, was initially underdiagnosed. 

Pulmonary symptoms, less common in MPA, affect 30-50% of patients, with varying patterns from focal infiltrates to diffuse IAH [5]. Approximately 25% of cases lack hemoptysis but exhibit a sudden drop in hemoglobin level with respiratory distress, strongly suggesting IAH [6]. When suspected, diagnosis is usually confirmed through analysis of bronchoalveolar lavage fluid showing a hemorrhagic appearance or hemosiderin-laden macrophages. Initial imaging in our case indicated infectious pneumonia, and MPA typically displays diffuse, bilateral ground-glass opacities, reflecting hemorrhagic alveolitis. However, the progressive and insidious nature of the disease can cause areas of IAH to appear consolidative when alveoli are filled with blood [7]. The use of oral anticoagulant and thrombocytopenia in our case may have contributed to this appearance.

A recent retrospective multicenter study, involving 368 patients with MPA from the French Vasculitis Study Group Registry, revealed a 20.6% incidence of cardiovascular involvement, primarily manifesting as heart failure and pericarditis [8]. Cardiac rhythm abnormalities, notably atrial fibrillation, and occasionally conduction issues, ranging from asymptomatic bundle branch block to complete AVB, were observed. Autopsy findings in some cases showed infarctions of the atrioventricular node or bundle of His and arteritis of the supplying arteries, along with one instance of granulomas in the interventricular septum [9,10]. Overall, complete AVB cases are considered exceptional in MPA, with only one reported case so far occurring in an elderly patient with multiple cardiovascular comorbidities [11]. In the presented case, AVB onset coincided with the MPA diagnosis during the active inflammatory phase of the disease. A retrospective analysis of 2371 patients with AAV demonstrated a higher prevalence of cardiovascular events in the year leading up to the AAV diagnosis, peaking one month before diagnosis and persisting during the first three months post-diagnosis [12]. This suggests a strong likelihood of a significant association between MPA and the observed AVB case.

Autoimmune diseases occur in 10-20% of MDS cases, mostly systemic medium and large vessel vasculitis (e.g., polyarteritis nodosa), connective tissue diseases, and neutrophilic dermatosis [13]. ANCA positivity is also more common in MDS patients, although it is not often associated with vasculitis symptoms, and therefore, systematic ANCA testing isn’t recommended [14]. In a recent retrospective multicenter study involving 2244 patients with hematologic malignancy, ANCA-associated vasculitis was estimated to occur in 0.37-1.6% of patients, often preceding or following the vasculitis diagnosis by approximately six months. In this series, MPA was the primary type of vasculitis, and 16 cases could be identified, of which 38.5% were MDS. Such a combination had a poor prognosis, with a median survival of three years, much lower than in isolated AAV [15]. Our patient had a confirmed MDS (supported by cytopenias, cytologic abnormalities, and clonal mutations). Interestingly, leukocytosis with circulating myeloid precursors and hepatomegaly combined with negative BCR-ABL transcript evoke a myelodysplastic/myeloproliferative neoplasm (MMN). TET2 and especially CBL mutations are more frequent in these syndromes [16,17]. However, the onset of leukocytosis was too rapid, with missing criteria for any entity of the MMN spectrum described so far; hence, the diagnosis remains MDS [18]. Nevertheless, we hypothesize that our patient’s molecular profile might have been responsible for the important observed leukocytosis. To this date and our knowledge, there isn’t any reported case of myelodysplastic/myeloproliferative syndrome associated with AAV.

## Conclusions

This case highlights the rare co-occurrence of MDS and MPA, with the unexpected complication of AVB. The delay in identifying pneumo-renal syndrome is regrettable. It underscores the importance of systematically assessing urinary sediment and proteinuria in patients with acute renal insufficiency and radiological pneumopathy. In such cases, considering AAV is essential. Our patient’s specific hematological condition combined with the initial unilobar presentation might have contributed to a presentation that more closely mimicked infectious pneumonia. Early intervention with immunosuppressants could have altered the course of this fatal IAH episode, although the overall prognosis would have remained challenging due to the extensiveness of the disease, infection risks, advanced age, and underlying hematological issues.
